# Cases of Abnormal Fœtuses, with Remarks

**Published:** 1838-08-15

**Authors:** 


*Cases of Abnormal Fœtuses, with Remarks, by Drs.* Lippincott *and* Warrington.

To the Editors of the Medical Examiner.

From my friend, Dr. Henry Lippincott, of Fallsington, Bucks county, I have received the following notice, to which he requested me to add any views I might entertain on the subject of the influence of the imagination on the condition of the fœtus in utero, with a desire that I would hand it over to you for insertion in the Medical Examiner.

"***** was advanced two and a half months in her pregnancy with her twelfth child, when she was exceedingly frightened by one of her sons having one of his hands dreadfully mangled and lacerated by the discharge of a gun passing through it, and tearing a part of it away; in this dreadful state he came directly and unexpectedly to her when alone, her husband being absent from home. The shock quite overcame her, and she became almost frantic with grief, and so continued for some length of time, with the dreadful ideas of death, locked-jaw, mortification, &c. &c. The boy, however, gradually recovered after a long time, and in twenty-nine and a half weeks from the day the accident happened, she was confined with a still-born son, of the common size, much deformed in several respects, having the appearance (I did not make a post-mortem examination) of a twin brain, with a very singular mark on the crown; had not any nose, but instead an opening void of any substance whatever; neither any palate; the upper jaw deficient only at the condyles; six fingers on each hand, and six toes on each foot; did not appear to be any opening in the penis like a urethra; neither were there any testes. The naval cord was also very strangely formed by having round and large globules about it, with the skin on parts of the body elevated by water, which is not unusual with still-born infants. During this woman's previous pregnancies, no particular fright occurred, neither are any of the former eleven children marked in any way whatever. She is aged about forty, and enjoys excellent health; is of a portly, robust frame."

Although I have met with several cases of deformity, or monstrosity, which the mothers or their friends were ready enough to refer to causes, I have hitherto not considered their reasons sufficiently satisfactory to enable me to form an opinion, that the irregularities in question, were consequences of any causes operating upon the imagination of the mother. I have frequently known mothers disappointed in not finding marks, &c., upon their children, because they had had great "longings, had been frightened," &c. I give below, an instance of a coincidence, in a case with which I was well acquainted.

Mary Risden, æt. about thirty-three, was delivered in Tenth mo., (October,) 1823, in the village of Colestown, New Jersey, of a fine, vigorous boy, apparently well formed, except the absence of the fore arms, and the pelvic extremities. The child grew finely, and lived ten months, when it died of croup. At the extremity of each arm, there was a prominent cicatrix, as though it had been amputated some months before; there was a tubercle (similar to that presented at the umbilicus) over the supposed situation of each acetabulum; the lower end of the abdomen terminated in a slightly obtuse point; there were no other peculiarities externally. The post mortem examination was made by Drs. J. J. Spencer and Stokes, of Moorestown, N. J., and myself. The os humeri of one side terminated, about four-fifths of its normal length, in a small tubercle, like that at the extremity of the fingers; the other humerus tapered towards its extremity, and terminated in a single condyle. The pelvis was normal, except that, in place of the acetabula, there were smooth, oval, little processes of bone. No attention was paid to the arrangement of the muscles of the parts, which I very much regret. It possessed the free use of its arms, as was manifest whenever its attention was attracted by any object presented to its sight.

The wise, cause-accounting neighbours declared, that this monster resulted from the sight of bull-frogs, the limbs of which had been amputated, for the purpose of supplying her with food; but, while attending upon this poor woman, during a subsequent parturition, in Fifth month, (May,) 1829, she informed me that, when about four and a half months advanced with her other child, she was suddenly surprised, whilst catching poultry for market, late one evening, by being seized at the elbows by her brother-in-law, and so much frightened, that she fell prostrate, and could not move her limbs for several days after. Instead, therefore, of the sight of the maimed frogs, the flesh of which she rarely ate, she attributed the defects in her child to the alarm she received, and the impressions made upon her system at the time. The children, born previous to this period, were well formed, as was that of which I delivered her, at the time of last date, (1829.) I give the above as a fact, which I have witnessed, accompanied with the opinions of the mother and her neighbours, but withhold my own, till I can meet with more satisfactory evidences of cause and consequence.

Yours, respectfully,

Joseph Warrington, 229 Vine st.

*Seventh month 18th*, 1838.

